# Biotic- and abiotic-driven variations of the night-time sap flux of three co-occurring tree species in a low subtropical secondary broadleaf forest

**DOI:** 10.1093/aobpla/ply025

**Published:** 2018-04-20

**Authors:** Qian Wang, Jianguo Gao, Ping Zhao, Liwei Zhu, Lei Ouyang, Guangyan Ni, Xiuhua Zhao

**Affiliations:** 1 Key Laboratory of Vegetation Restoration and Management of Degraded Ecosystems, South China Botanical Garden, Chinese Academy of Sciences, Guangzhou, PR China; 2 College of Resources and Environment, University of Chinese Academy of Sciences, Beijing, PR China; 3 Institute of Biodiversity Science, Fudan University, Shanghai, PR China; 4 Guangdong Provincial Key Laboratory of Applied Botany, South China Botanical Garden, Chinese Academy of Sciences, Guangzhou, PR China

**Keywords:** Inter- and intraspecific variations, night-time water use, sap flux density, stem green tissue, water recharge

## Abstract

Although several studies on the night-time water use of different plant species have been reported, comparative studies under the same climatic conditions of a region are scarce. This study aimed to analyse the inter- and intraspecific variations in night-time water use in relation to environmental factors and to tree morphological features to understand and elucidate the possible underlying mechanisms. The sap flow of three co-occurring tree species in a low subtropical secondary broadleaf forest in South China was monitored using Granier-style sap flux sensors. All examined environmental factors except wind speed exerted significant influence on the daytime sap flows of *Schima superba*, *Castanopsis hystrix* and *Michelia macclurei*, but the impacts of all factors, including wind speed, on the night-time sap flux were trivial. These results indicated that sap flow was mainly used for water recharge at night. The morphological features of the trees, except tree height, significantly affected the daytime water use, but no morphological features significantly affected the night-time water use. We found that night-time water recharge was strongly affected by the maximum flux density. A principal component analysis showed that there were more intraspecific than interspecific variations in water transport. The results also revealed that the night-time water use and the percentage of night/day (*Q*_n_/*Q*_d_) of photosynthetic stem species (*C. hystrix* and *M. macclurei*) were greater than those of non-photosynthetic stem species (*S. superba*).

## Teaser

Aiming to analyse the inter- and intraspecific variations in night-time water use, we used Granier-style sap flux sensors to monitor the sap flow of three co-occurring tree species in a low subtropical secondary broadleaf forest in South China. A principal component analysis showed that there were more intraspecific than interspecific variations in water transport. The results indicated that sap flow was mainly used for water recharge at night, and the night-time water use and the percentage of night/day (*Q*_n_/*Q*_d_) of photosynthetic stem species (*Castanopsis hystrix* and *Michelia macclurei*) were greater than those of non-photosynthetic stem species (*Schima superba*).

## Introduction

The leaf stoma is the site where water is transpired into the ambient air, namely, transpiration (*E*). It has long been reported that although leaf stomata are open during the day, they are also partially open during the night (Darwin 1898; Gindel 1970; Snyder *et al.* 2003). Stomatal opening is attributable to genetics and is closely related to external environmental factors. It has been reported that *Ouratea hexasperma* has reduced stomatal conductance (*g*_s_) in nitrogen-rich environments, so the amount of water used greatly declines at night (Scholz *et al.* 2007). This suggests that the plant’s night-time water use may be related to nitrogen uptake and can be affected by available soil nitrogen. Another study has shown that soil nitrogen limitation did not affect the night-time transpiration (*E*_n_) or water loss of two *Populus* species, which mainly showed effects related to soil moisture conditions (Howard and Donovan 2010). The fact that plants lose water at night but do not acquire nutrients appears to be a paradox for night-time stomatal opening. However, Dawson *et al.* (2007) studied 18 trees and shrubs in seven vegetation types and found that night-time transpiration or a certain degree of *g*_s_ were related to soil moisture fluctuation. They inferred that night-time stomatal opening was conducive to the assimilation of carbon by plants in the early morning of the next day. It may help plants absorb soil nutrients and transport oxygen to all parts of the parenchyma. Predawn stomatal opening may increase plant photosynthesis and probably helps plants take up and utilize nitrogen (Snyder *et al.* 2008). However, studies have also shown that if a plant grows in moist soil conditions, the positive effects of night-time transpiration and water use on plant growth and nutrient absorption are trivial (Howard and Donovan 2007, 2010; Christman *et al.* 2009). Currently, existing evaluation on the ecophysiological significance of night-time transpiration or water loss at night is inconsistent. Thus, more experiments are needed to elucidate the ecophysiological significance of night-time water use by plants.

Night-time water loss differs among plant species. However, few studies have focused on its ecological significance (Marks and Lechowicz 2007; Phillips *et al.* 2010). It must not be neglected that paradox on night-time sap flow’s ecological significance may be related to different methods used among studies (Forster 2014). Forster (2014) found that the heat ratio method (HRM) and the thermal dissipation probe (TDP) method had significantly higher Q_n_/Q (nocturnal sap flow/total daily sap flow) than the heat balance method. Normally, plants with vigorous growth at the top canopy have larger night-time sap fluxes in order to maintain sufficient water content at night (Daley and Phillips 2006; Marks and Lechowicz 2007). Daley and Phillips (2006) studied the night-time water-use characteristics of paper birch (*Betula papyrifera*), red oak (*Quercus rubra*) and red maple (*Acer rubrum*) using Granier-style sap flux sensors and found that the night-time water use of paper birch accounted for 10 % of the total sap flow, which was greater than that of the other two species. They thought that paper birch risks water loss through night-time transpiration because that strategy may provide it with an ecological advantage, enabling the trees to maximize photosynthesis in the early morning of the following day, thus supporting rapid growth. Observations on the *E*_n_ of 21 Canadian temperate deciduous tree species seedlings showed that nocturnal transpiration functioned to sustain carbohydrate export and other processes driven by dark respiration. It was concluded that this function was most important in fast-growing and shade-intolerant tree species (Marks and Lechowicz 2007). Although numerous studies on the night-time water use of different plant species have been reported, comparative studies under the same climatic conditions in a single region are scarce. Nevertheless, such comparisons would help in understanding the water-use strategies and mechanisms of co-occurring plant species (Phillips *et al.* 2010; Zeppel *et al.* 2010).

It is reported that respiration in wood parenchyma is limited by insufficient oxygen supply (Gansert *et al.* 2001). Wittmann and Pfanz (2014) speculated that bark and woody tissue photosynthesis could be important for preventing low oxygen limitation of respiration in dense and metabolically active tissues. Pfanz *et al.* (2002) also found that stem photosynthesis can generate oxygen to relieve anoxia in stem wood. Sap flow is an alternative pathway for oxygen delivery (Gansert 2003), since substantial oxygen can be delivered to xylem parenchyma cells in the aqueous state through sap flux (Mancuso and Marras 2003). Gansert (2003) also revealed that oxygen concentrations in the xylem reached minimum values after sundown. Night-time sapflux may play a vital role in delivering oxygen during this period of the diurnal cycle when oxygen concentrations can be critically low (Daley and Phillips 2006).

Accordingly, this research selected *Schima superba*, which does not show stem photosynthesis, and *Castanopsis hystrix* and *Michelia macclurei*, which have photosynthetic stems, to investigate the intra- and interspecific variations in night-time sap flow. The following questions were addressed. (i) Are there significant night-time intra- and interspecific water-use variations? (ii) How do biotic and abiotic factors drive the sap flow at night? (iii) Would the tree species with green bark on the stems (stem photosynthesis) have a higher proportion of night-time sap flow to the total daily sap flow than those without green bark?

## Methods

### Site description and studied tree species

The experimental site for this research is located in the Heshan National Field Research Station of Forest Ecosystem, Chinese Academy of Sciences, Guangdong Province, China (112°54′E, 22°41′N). The station has an average elevation of 80 m. This region is characterized by a subtropical monsoon climate, the annual average evaporation is 1600 mm, the annual precipitation is 1700 mm, and the annual average temperature is 21.7 °C. The mean lowest and highest temperatures are 13.1 °C in January and 28.7 °C in July, respectively. The annual accumulated temperature above 10 °C is 7597.2 °C, and the annual solar radiation is 4350.5 MJ m^−2^. Hydrothermal resources are abundant in this region, where the climax community, the subtropical monsoon evergreen broad-leaved forest, has been seriously impacted by human disturbance and soil erosion, which has resulted in degraded vegetation (barren, hilly grasslands) (Fu *et al.* 2011). Since the mid-1980s, a vegetation restoration campaign has been in place to recover the barren, hilly grasslands with fast-growing pioneer tree species. After more than 30 years, the pioneer communities have developed into various types of secondary broadleaf forests.

We conducted the field experiment in a secondary broadleaf forest that was developed from the revegetation with three dominant co-occurring tree species, *S. superba*, *C. hystrix* and *M. macclurei*. The three species are very common, native and pioneering in the forests of this area. A total of three trees of each species were chosen for this experiment, and they were all in close proximity to each other. The morphological features of the trees are listed in Table 1. The forest contains an acrisol soil with a topsoil (0–20 cm) pH of 4.26, an organic carbon matter content of 24.2 g kg^−1^, a total nitrogen content of 1.2 g kg^−1^ and an available phosphorus content of 2.4 mg kg^−1^. During the experimental period, the soil water content was 28.40 % ± 0.81 and remained fairly constant, probably because of the dense forest canopy.

**Table 1.** Morphological features of the three co-occurring tree species: *Schima superba* (trees 1, 2 and 3), *Castanopsis hystrix* (trees 4, 5 and 6) and *Michelia macclurei* (trees 7, 8 and 9). DBH: diameter at breast height; *A*_s_: sapwood area; *H*: tree height; *H*_u_: stem height under branch; *S*_c_: canopy size.

Tree species	Tree number	DBH (cm)	*A*_s_ (cm^2^)	*H* (m)	*H*_u_ (m)	*S*_c_ (m^2^)	Canopy position
*S. Superba*	Tree 1	13.4	118.1	4.73	4.0	7.8	Mid-canopy
	Tree 2	11.0	79.1	6.6	4.3	12.9	Mid-canopy
	Tree 3	13.1	112.8	6.85	3.6	15.5	Understory
*C. hystrix*	Tree 4	12.9	97.8	11.49	6.4	7.2	Canopy
	Tree 5	12.1	86.5	12.35	4.7	19.0	Canopy
	Tree 6	12.9	97.8	9.39	3.9	23.5	Canopy
*M. macclurei*	Tree 7	18.2	201.9	11.71	5.5	29.6	Canopy
	Tree 8	11.6	81.8	6.73	3.9	11.7	Canopy
	Tree 9	18.0	197.5	15.07	3.6	27.3	Canopy

#### Sap flux density (*J*_s_).

Stem sap flow measured by the thermal technique can be used for characterizing the transpiration of whole trees and stands. In this study, home-made Granier’s sensors (TDPs) were directly inserted into the xylem of the nine sample trees for sap flow monitoring (Granier 1987). The TDP sensors, each consisting of a pair of stainless steel probes and each 20 mm long and 2 mm in diameter, were inserted approximately 10–15 cm apart along the axis of the hydro-active xylem. The upper probe was heated by a constant power of 0.2 W with a DC supply of 120 mA, while the lower probe remained unheated.

As the upper probe is steadily heated, the sap flow will carry part of the heat upward, thus reducing the temperature difference between the upper and lower probes. When transpiration does not occur, or when there is no sap flow, a maximum temperature difference exists between the two probes. The instantaneous temperature differences between the two probes yielded a voltage value that was recorded by a data collection instrument (Delta-T logger, DL2e, UK). The data were measured every 30 s and stored as 10 min averages (Wang *et al.* 2012; Zhao *et al.* 2013). Finally, the sap flux density (g m^−2^ s^−1^) was calculated according to the following formula:

$$J_{s}=119\times {\left(\frac{\Delta T_{m}-\Delta T}{\Delta T}\right)}^{1.231}$$ (1)

where Δ*T*_m_ is the temperature difference between the two probes obtained under zero flow conditions and Δ*T* is the instantaneous temperature difference. The ‘zero baseline’ was set on nights when atmospheric vapour pressure deficit (VPD) was zero or almost approaching zero for several hours, so that there was no driving force for sap flux. Zero baselines on these nights were set as late into the night as dew point persisted, after baselines reached stable values. Moreover, Δ*T*_m_ was determined separately for each tree over 7 days to avoid the underestimation of nocturnal sap flux (Lu *et al.* 2004). We converted the voltage value into *J*_s_ by applying the Baseliner software developed by Dr Yavor Parashkevov in the Nicolas School of Environment and Earth Science at Duke University, USA.

The xylem wood of *S. superba* and *C. hystrix* is diffuse-porous, while that of *M. macclurei* is semi-diffuse-porous. According to a previous study, the radial sap flux density does not vary significantly in diffuse-porous wood (Clearwater *et al.* 1999). We calculated the radial variation of the *J*_s_ along the sapwood depth of *M. macclurei* following Pataki *et al.* (2011) and found that there was non-significant variation in *J*_s_. Therefore, the whole-tree water use per day can be directly calculated by summing the sap flux density multiplied by the sapwood area (*A*_s_):

$$Q=\sum^{\text{​}}(J_{s}\times A_{s}\times t)\;(\text{kg per day})$$ (2)

where ‘*t*’ means 600 s (10 min), for the data being stored as 10 min averages. *Q* means the total sum of water use, the daytime and night-time lengths range from 0600 to 1900 h and from 1900 to 0600 h, respectively (Zhao *et al.* 2013).

#### Sapwood area, diameter at breast height, tree height and canopy size.

To avoid damage to the trees monitored for sap flow trees during the measurement of sapwood area, we selected 16 *S. superba*, 17 *C. hystrix* and 21 *M. macclurei* trees of different diameter classes in the neighbouring area surrounding the experimental plot and obtained tree core samples using an increment borer (diameter 5 mm). The boundary between the sapwood and the heartwood was identified visually. With these data, an exponential relationship between the diameter at breast height (DBH) and the sapwood area (*A*_s_) was established:

$$A_{\text{s}}=\;a\text{DB}{\text{H}}^{b}$$ (3)

where *a* and *b* are coefficients obtained through a non-linear regression analysis. The regression equations for *S. superba*, *C. hystrix* and *M. macclurei* were: *A*_s_ = 0.7248 × (DBH)^1.9418^ (*R*^2^ = 0.992, *P* < 0.0001), *A*_s_ = 0.7295 × (DBH)^1.9133^ (*R*^2^ = 0.988, *P* < 0.0001) and *A*_s_ = 0.6465 × (DBH)^2.0054^ (*R*^2^ = 0.993, *P* < 0.0001), respectively. These equations were used to determine the sapwood area of the sample trees for the sap flow measurement. The DBH was directly measured at 1.3 m above the ground using a DBH tape measure. The tree height (*H*) was measured using a Tandem-360R/PC type altimeter (Sunto, Finland) according to a trigonometric formula, and the stem height under branch (*H*_u_) was determined using the same method as used for the tree height measurement. By assuming an elliptical shape, the canopy size (*S*_c_) was calculated by an elliptic equation after measuring the major (*d*_1_) and minor (*d*_2_) axes of the canopy of each tree:

$$S_{c}=\frac{\pi \times d_{1}\times d_{2}}{4}$$ (4)

#### Wood density, sapwood water content and saturated water content.

For each species, we drilled the sapwood from six trees outside the experimental plot using the same increment borer mentioned in the previous section. The drilled wood cores were wrapped with a wet towel immediately after sampling and then placed in sealed plastic bags. They were quickly brought to a nearby laboratory in the Heshan Station and weighed using an electronic balance (Shinko, Japan) with an accuracy of 0.0001 g. The wood cores were soaked overnight in water for 24 h, and the saturated fresh weights were measured after removing the moisture on the core surface. Finally, the wood cores were dried to a constant weight to obtain a dry weight. The wood density (*W*_d_), sapwood water content (*W*_w_) and saturated water content (*W*_sw_) were calculated as follows (Borchert 1994; Gao *et al.* 2015):

$$W_{\text{d}}=\text{ dry mass/fresh volume}$$ (5)

$$W_{\text{w}}=\text{ 1}00(\text{fresh mass}-\text{dry mass})/\text{fresh mass}$$ (6)

$$W_{\text{sw}}=\text{ 1}00(\text{saturated mass}-\text{dry mass})/\text{dry mass}$$ (7)

#### Meteorological data.

Data on the photosynthetically active radiation (PAR), air temperature (*T*_a_), air humidity (RH) and wind speed (m s^−1^) were recorded at a meteorological station approximately 100 m away from the experimental plot. The wind speed data were recorded once every 2 min and then averaged hourly, and the other meteorological data were averaged hourly. We calculated the VPD (kPa) by combining the air temperature and the air humidity according to Campbell and Norman (1998):

VPD= a·exp(b·T/(T+c))(1−RH) (8)

where *a*, *b* and *c* are fixed parameters that are 0.611 kPa, 17.502 (unitless) and 240.97 °C, respectively.

#### Data analysis.

The statistical analyses of the inter-species variations of night-time water transport were performed via one-way ANOVA using Predictive Analytics Software (PASW, IBM, USA). The least significant difference at *P* = 0.05 denoted significance. To illustrate the main factors affecting water use, we carried out a principal component analysis (PCA) using tree morphological factors: wood density (*W*_d_), sapwood water content (*W*_w_) and saturated water content (*W*_sw_) and using water-use characters: the total daytime water use (*Q*_d_), the total night-time water use (*Q*_n_), the maximum daytime sap flux density (*J*_smax,d_) and the maximum night-time sap flux density (*J*_smax,n_). The tree structural characteristics are considered important factors affecting water use (Wang *et al.* 2007, 2012; Zhou *et al.* 2012; Zhao *et al.* 2013) and can be described by diameter at breast height (DBH), tree height (*H*), height under branch (*H*_u_), sapwood area (*A*_s_) and canopy size (*S*_c_). The relationships between the environmental factors and the sap flux were fitted by linear and non-linear regression analysis methods. A paired *t*-test was used to compare the daytime vs. the daytime plus night-time water use of the selected trees because there are distinct tree-to-tree variations (Zhou *et al.* 2012).

## Results

### Inter- and intraspecific variations in daytime and night-time sap flux and the effects of tree morphological features

**Figure 1.**
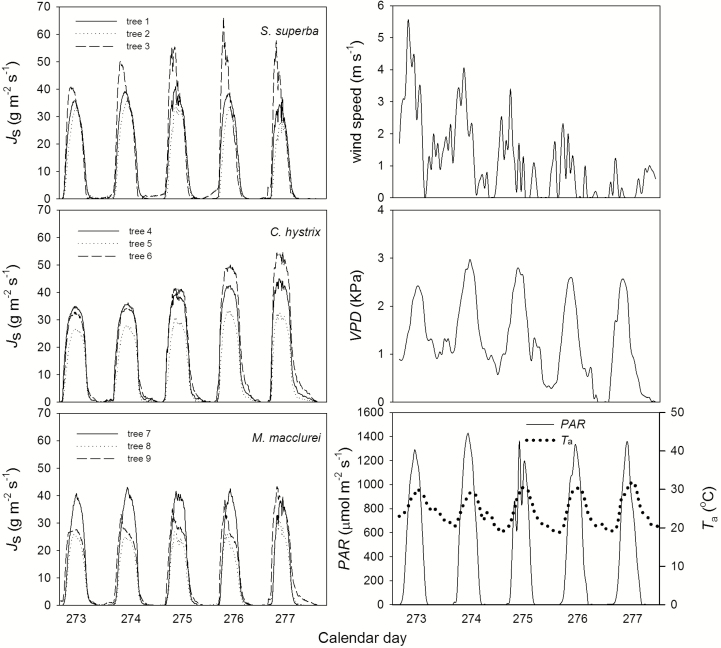
Daily changes in sap flux density (*J*_s_) in the three examined co-occurring tree species; PAR, VPD, *T*_a_ and wind speed over five consecutive days during the experimental period.

**Figure 2.**
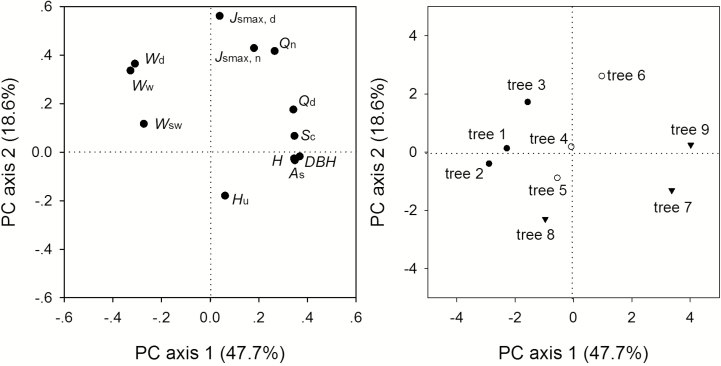
Principal component analysis (PCA) on the factors affecting the night-time and daytime sap flow (left) and the clustering of the whole-tree water use of the nine sap-flow-monitored trees of the three species (right): the dots, circles and triangles denote *Schima superba*, *Castanopsis hystrix* and *Michelia macclurei*, respectively.

The sap flow data as monitored for five consecutive sunny days (calendar days 273–277 in 2013) were selected for the analysis. The diurnal course of the sap flux density of each tree was a single peak (Fig. 1). The daytime maximum sap flux density (*J*_smax,d_) was 40.9, 35.6 and 65.7 g m^−2^ s^−1^ for trees 1, 2 and 3 (*S. superba*), respectively, and was 44.9, 33.5 and 54.8 g m^−2^ s^−1^ for trees 4, 5 and 6 (*C. hystrix*), respectively. The *J*_smax,d_ of *M. macclurei* was comparatively lower, at 43.0, 31.0 and 43.2 g m^−2^ s^−1^ for trees 7, 8 and 9, respectively. The night-time maximum sap flux density (*J*_smax,n_) of *C. hystrix* was the highest, at 4.2–13.7 g m^−2^ s^−1^ and averaging 8.3 g m^−2^ s^−1^, the *J*_smax,n_ of *M. macclurei* was 2.7–7.7 g m^−2^ s^−1^ (average of 4.4 g m^−2^ s^−1^) and that of *S. superba* was 2.1–2.7 g m^−2^ s^−1^ (average of 2.5 g m^−2^ s^−1^) (Fig. 1). The sap flux densities of trees 2, 5 and 8 were the lowest for each of the three tree species, which was verified by the PCA results because they were located in the third quadrant (Fig. 2, right). The night-time water use by *S. superba* was 0.088, 0.105 and 0.218 kg for trees 1, 2 and 3, respectively, averaging 0.137 kg, and the CV (coefficient of variation) was 44.1–90 % (averaging 61.8 %). The average nocturnal water use of *C. hystrix* was 0.510, 0.193 and 0.852 kg for trees 4, 5 and 6, respectively, and the CV was 51.2–72.2 % (averaging 63.2 %). The night-time water use of trees 7, 8 and 9 was 0.226, 0.096 and 0.754 kg, respectively, and the CV was 67.7–140 %, with an average of 88.3 %. The interspecific CV was 4.6–32.5 % (averaging 17.9 %). The intraspecific variation in water use was clearly much larger than the interspecific variation, which is in agreement with the observations by Phillips *et al.* (2010).

**Figure 3.**
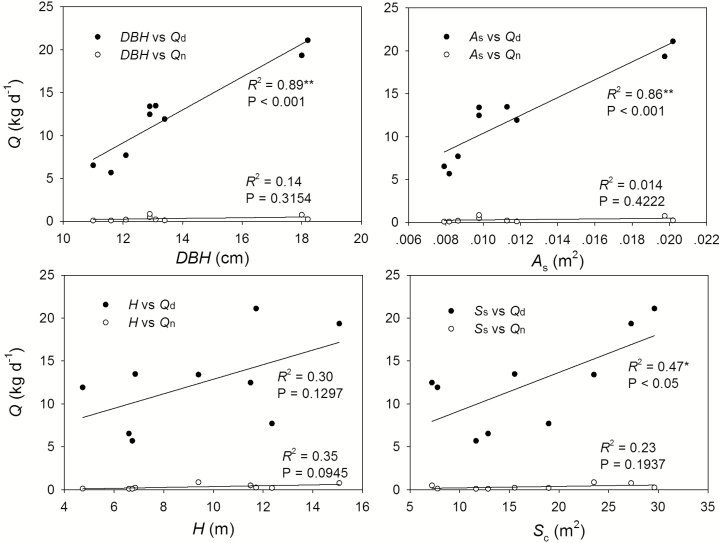
Regression analysis of the tree morphological features (DBH, *A*_s_, H and *S*_c_) and the total daytime (*Q*_d_) and night-time (*Q*_n_) water use (*P < 0.05; **P < 0.001).

Since the measured trees grew near each other in the experimental plot, we pooled all the data together irrespective of tree species and performed PCA on the hydraulic architecture and wood water content, as well as the daytime and night-time water use and maximum sap flux densities. PC1 and PC2 explained 47.7 and 18.6 %, respectively, and together, they explained 66.3 % of the variation in water use (Fig. 2, left). *Q*_d_, *Q*_n_, *J*_smax,d_ and *J*_smax,n_ were located in the first quadrant; *W*_d_, *W*_w_ and *W*_sw_ were distributed in the second quadrant; and the tree morphological features except for *S*_c_ were scattered in the fourth quadrant. The relationship between the tree water use and the tree morphological features (DBH, *H*, *H*_u_ and *A*_s_) was illustrated by regression method, which showed that daytime water use was significantly affected by the tree morphological features except *H*. For example, DBH, *A*_s_, *H* and *S*_c_ can explain 89.0, 86.0, 30.0 and 47.0 % of the variations in daytime water use, respectively. Except for *H*, all tree features significantly influenced *Q*_d_. However, the tree morphological features did not significantly affect the night-time water use (Fig. 3).

#### The environmental factors in relation to the sap flux variation.

As shown in Fig. 1, the PAR presented a single diurnal peak during the experiment. It rose from 20 μmol m^−2^ s^−1^ at 0700 h to 1427 μmol m^−2^ s^−1^ at 1300 h, and then declined to 0 μmol m^−2^ s^−1^ at 1900 h. The VPD is the main driver of sap flux and ranged from 0 to 3 kPa during the daytime. Its maximum was 2.97 kPa at 15:00 on calendar day 274. The VPD ranged from 0 to 1.5 kPa at night. The maximum wind speed was 5.5 m s^−1^ with an average of 1 m s^−1^. The atmospheric temperature (*T*_a_) showed a pattern similar to the PAR, averaging 24 °C, and the highest and the lowest temperatures were 31.8 and 18.6 °C, respectively.

**Figure 4.**
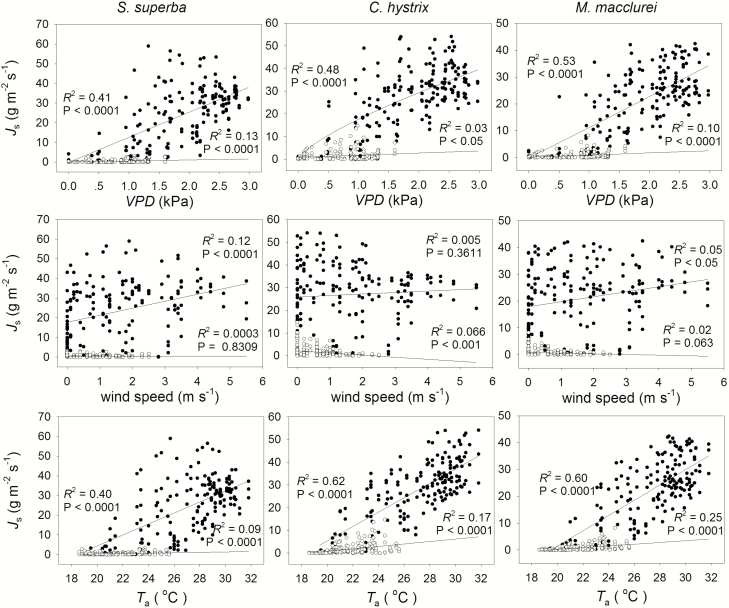
Environmental drivers and sap flux density. The black dots represent the driving environmental factors vs. the daytime sap flux density, while the circles denote the same driving factors vs. the night-time sap flux density.

A correlation analysis was performed to describe the impacts of the VPD, wind speed and air temperature on sap flux density (Table 2; Fig. 4). The VPD explained 41 % of the daytime and 13 % of the night-time sap flux variations in *S. superba*, which could be fitted by a linear equation. The correlation between the sap flux of *C. hystrix* and the VPD could be well fitted by a power equation (*R*^2^ = 0.48, *P* < 0.0001). Wang *et al.* (2007) found that the night-time sap flux density was significantly correlated with the VPD, but the correlation coefficient was low (*J*_s,n_ vs. VPD, *R*^2^ = 0.056 *P* < 0.05). They concluded that night-time sap flux had weak correlation with the VPD. Similarly, the VPD exerted only a very small effect on the night-time sap flux of *C. hystrix* (*R*^2^ = 0.03, *P* < 0.05). The effects of the VPD on the daytime sap flux of *M. macclurei* were non-linear (*R*^2^ = 0.53, *P* < 0.0001), but the VPD had a slight effect on the nocturnal sap flow (*R*^2^ = 0.1, *P* < 0.0001). The wind speed had little effect on the daytime sap flux density in *S. superba* (*R*^2^ = 0.12, *P* < 0.0001), while it had negligible effects on the other two species during both the day and the night. The atmospheric temperature explained 40, 62 and 60 % of the daytime sap flux variation and 9, 17 and 25 % of the night-time sap flux variations for *S. superba*, *C. hystrix* and *M. macclurei*, respectively. Only a small effect of the temperature on the tree water use at night was observed.

**Table 2.** Equations from regressions between day/night sap flux density (*J*_s,d_/*J*_s,n_) and environmental drivers (VPD, wind speed (WS), *T*_a_) of three co-occurring tree species: *Schima superba*, *Castanopsis hystrix* and *Michelia macclurei*. Non-linear relationships are represented in bold.

*S. superba*	*C. hystrix*	*M. macclurei*
*J*_s,d_ = 12.8VPD − 0.16	***J***_**s,d**_ **= 17.68VPD** ^**0.73**^	***J***_**s,d**_ **= 11.17VPD** ^**1.03**^
*J*_s,n_ = 0.51VPD + 0.05	*J*_s,n_ = 0.93VPD + 0.89	*J*_s,n_ = 0.82VPD + 0.08
*J*_s,d_ = 3.56WS + 17.78	*J*_s,d_ = 0.64WS + 25.95	*J*_s,d_ =1.85WS + 17.93
*J*_s,n_ = 0.02WS + 0.40	*J*_s,n_ = −0.92WS + 2.13	*J*_s,n_ = −0.25WS + 0.81
*J*_s,d_ = 2.79*T*_a_ − 51.45	*J*_s,d_ = 3.17*T*_a_ − 58	*J*_s,d_ =2.85*T*_a_ − 55.51
*J*_s,n_ = 0.11*T*_a_ − 1.87	*J*_s,n_ = 0.54*T*_a_ − 10	*J*_s,n_ = 0.32*T*_a_ − 6.29

**Figure 5.**
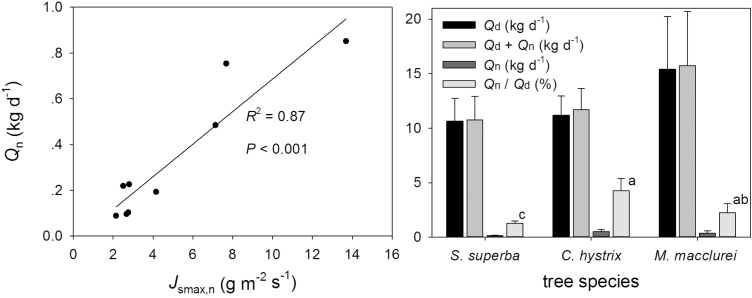
Regression analysis of the night-time water use (*Q*_n_) and the maximum night-time sap flux density (*J*_smax,n_) (left); comparative results of the tree water use among the three species. The different small letters denote significance at the 0.05 level (*P* < 0.05) (right), and the data are shown as the means ± SE.

 We found that the night-time water use was strongly affected by *J*_smax,n_, and this effect was irrespective of the tree morphological features when we pooled the measured data from all three tree species and analysed them together (*R*^2^ = 0.870, *P* < 0.001; Fig. 5, left). Among the three tree species, the highest total daily water use (*Q*_d_ + *Q*_n_) was observed in *M. macclurei*. The percentage of *Q*_n_/*Q*_d_ was highest in *C. hystrix* (Fig. 5, right), with an average of 4.2 %, which was significantly higher than that in *S. superba* (1.3 %, *P* < 0.05). It was also higher than that of *M. macclurei* (2.2 %), but this difference was not significant. The tree–tree paired *t*-test showed that there was no difference between the daytime (*Q*_d_) and the daytime plus night-time (*Q*_d_ + *Q*_n_) water use, indicating a trivial contribution by the night-time water use to the canopy transpiration. Therefore, *Q*_n_ could be neglected when evaluating the whole-tree total water use (Fig. 5, right).

## Discussion

### Night-time water use as recharge

Previous studies have reported that the major environmental factors affecting sap flow during the day are the PAR and the VPD, while the major factors at night are the VPD, wind speed and tree features (Oren *et al.* 1999; Zeppel *et al.* 2010; Rosado *et al.* 2012; Wang *et al.* 2012; Zhou *et al.* 2012). Rosado *et al.* (2012) observed a non-linear relationship between the sap flow-based transpiration and the VPD at a higher altitude site, which is consistent with our study on *C. hystrix* and *M. macclurei*. Unlike *S. superba*, *C. hystrix* and *M. macclurei* were top canopy species, and their high sensitivity to the VPD reflected their dominant positions in the community. This study demonstrated that the VPD, wind speed and temperature (*T*_a_) had very limited effects on the nocturnal sap flow (Fig. 4), implying a weak contribution of the sap flow to canopy stomatal transpiration during the night-time period.

**Figure 6.**
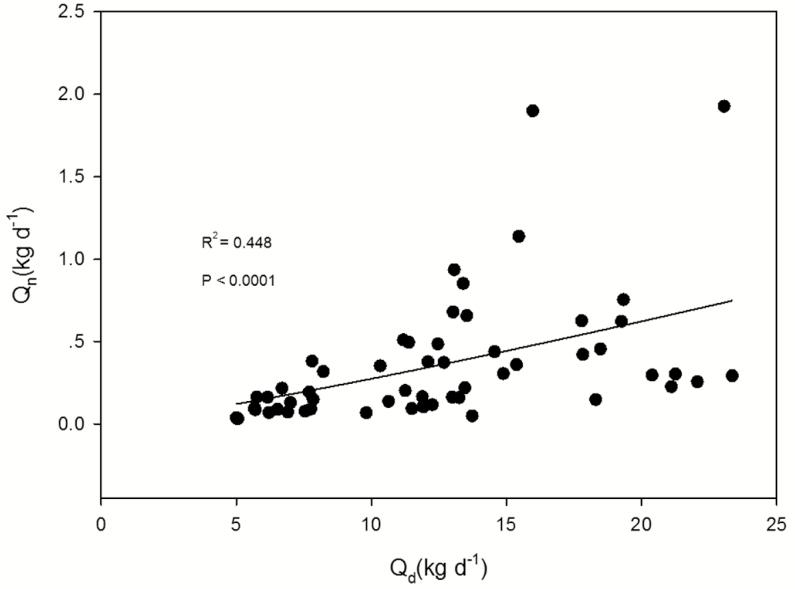
Regression analysis of the night-time water use (*Q*_n_) and the daily water use (*Q*_d_): *y* = 0.0078*x*^1.4110^ (*R*^2^ = 0.448, *P* < 0.0001).

Night-time sap flow is mainly used for either transpiration or replenishment of the water deficit. As the wind ventilates and alters the air status within and above the canopy, the VPD changes accordingly, in turn inducing variation in the leaf transpiration. By analysing the correlation between the sap flux density and the VPD and wind speed, the main function of the night-time water use can be illustrated if it is used for transpiration or recharge. If the VPD and wind speed do not explain the variation in night-time sap flux, the water use is considered to be mainly for recharge (Caspari *et al.* 1993; Benyon 1999; Daley and Phillips 2006; Wang *et al.* 2007). Phillips *et al.* (2010) selected two ‘perfect’ consecutive days to partition the transpiration and water recharge. Nevertheless, that method likely overestimates recharge and yields conservative estimates of *E*_n_, indicating the necessity for further evaluation of the methodology. Wang *et al.* (2007) found that the VPD and the wind speed affected the night-time sap flux in *Acacia mangium* trees. Although the night-time sap flux density was significantly correlated with the VPD and wind speed, the correlation coefficient was low (*J*_s,n_ vs. VPD, *R*^2^ = 0.056; *J*_s,n_ vs. wind speed, *R*^2^ = 0.014). Hence, they concluded that the night-time water use was mainly for recharge. Similarly, as described in the previous section, the night-time sap flux was weakly correlated with the VPD as well as the wind speed (Fig. 4). It can therefore be concluded that the nocturnal sap flow was mainly used for water recharge in our case, which is consistent with previous studies on *S. superba* (Zhou *et al.* 2012; Zhao *et al.* 2013), *A. mangium* (Wang *et al.* 2007), paper birch (*B. papyrifera*), red oak (*Q. rubra*) and red maple (*A. rubrum*) (Daley and Phillips 2006). In addition, based on the principle of water balance, there was a significant correlation between daily water use and night-time water use (*R*^2^ = 0.448, *P* < 0.0001) (Fig. 6), which indirectly supported the idea that the nocturnal sap flow was mainly used for refilling the tree hydraulic capacitance.

Previous studies have shown that the DBH is the main tree morphological feature that correlates with the whole-tree water use (Enquist *et al.* 1998; Meinzer *et al.* 2001). In a literature review, trees with a DBH ranging from 5 to 10 cm were reported to consume water at a rate of 10–150 kg per tree, while those with a DBH between 37 and 42 cm were found to use water at a rate of 52–349 kg per tree (Wullschleger *et al.* 1998). Wang *et al.* (2007) found that the nocturnal water use of *A. mangium* was strongly affected by the DBH, *H* and canopy size. Zhou *et al.* (2012) and Zhao *et al.* (2013) found that the night-time water use could be well explained by the DBH, *H*, canopy size, trunk and leaf biomass, and they also reported the seasonal differences of such effects by tree morphological features. In the fall, the night-time water use was mainly affected by the DBH, whereas in the winter, it was mainly affected by the tree height. Nevertheless, this is not always the case. In this study, all of the tree morphological features except for tree height significantly affected the daytime water use but had non-significant effects on the night-time water use, indicating that the tree morphological features could not be used to explain the night-time sap flux. As night-time sapflow is related with the refilling of tree hydraulic capacitance, there would be a stronger relationship of night-time sapflow with size-related traits, as bigger trees would potentially have a higher water storage capacity. However, we found size-related traits had insignificant effects on the night-time water use in this study. This might be due to the fact that nocturnal sap flow is mainly related to water deficit produced by the previous daytime transpiration (Fig. 6). Moreover, we found that the night-time water use was very significantly affected by *J*_smax,n_ (Fig. 5, left). Water consumed during the day will be supplemented at night; furthermore, the trees of larger daytime water use would potentially have a higher *J*_smax,n_ for water recharge, and *J*_smax,n_ significantly affected night-time water use. This circular relation indirectly supported the idea that night-time water use is considered as recharge.

#### Conservative night-time water use.

Plants always adjust their stomatal apertures to maximize photosynthesis while minimizing the consequences of excessive water loss (Cowan and Farquhar 1977; Héroult *et al.* 2013). Normally, plants lose water driven by variation in the VPD. The nocturnal VPD is lower than in the daytime, and only a small amount of plant water loss occurs at night. The night-time sap flux was found to be 5 % of the total water use in a *Eucalyptus grandis* plantation (Benyon 1999). Phillips *et al.* (2010) studied the water-use characteristics of eight *Eucalyptus* species in Australia and found that the night-time sap flow accounted for 5–7 % of the daily total. The night-time water use of *Eucalyptus* trees is comparatively low, and their intraspecific night-time water use varies more than their interspecific night-time water use. They believed that *Eucalyptus* trees had strong control over night-time water loss. Zeppel *et al.* (2010) reported that the mean seasonal nocturnal sap flow was 6–8 % of the 24-h flux across three seasons (spring, summer and winter) in the co-occurring evergreen species *Eucalyptus parramattensis* and *Angophora bakeri*. The nocturnal water use accounted for 4–6 % of the total annual stand-scale transpiration, indicating that *Q*_n_ can be ignored when estimating the annual stand transpiration (Zhao *et al.* 2011). Zhou *et al.* (2012) found that the night-time water use was 2–8 % of the whole-tree transpiration for *S. superba*, and significant seasonal variations existed, namely the night-time sap flow was significantly higher in the dry season than in the wet season. Our study demonstrated that the night-time water use of *C. hystrix* was the highest, as was the percentage of *Q*_n_/*Q*_d_ (Fig. 5, right), which averaged 4.2 %. The percentage of *Q*_n_/*Q*_d_ of *C. hystrix* was significantly higher than that of *S. superba* (1.3 %) and *M. macclurei* (2.2 %) (*P* < 0.05). The contribution of night-time water use to canopy transpiration was verified by a paired *t*-test, which showed that there was no difference between the daytime (*Q*_d_) and the daytime plus night-time (*Q*_d_ + *Q*_n_) water use, indicating that night-time water use was negligible. Lower plant water use at night is the result of adaptation to the environment. The trivial nocturnal water use observed in the three co-occurring woody plants in this study indicates that these native pioneer species have developed a conservative water-use strategy at night.

#### Ecological implications of night-time water use.

**Figure 7.**
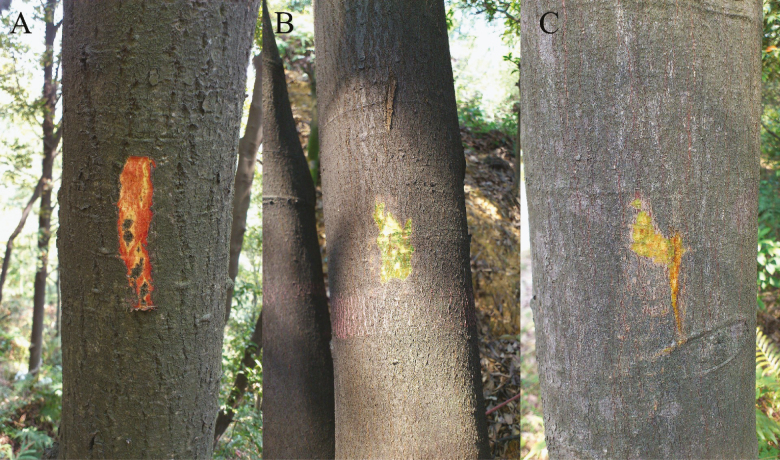
Stem barks of the studied tree species with and without chlorenchyma. (A, B and C denote *Schima superba*, *Castanopsis hystrix* and *Michelia macclurei*, respectively.)

Greater relative growth rates tend to correspond to higher respiration rates for fast-growing species, whereas slow-growing species spend more energy on nutrient uptake (Poorter *et al.* 1991). The photosynthesis by the stems of the shrub *Myrica cerifera* was found to assist in the successful invasion of new habitats due to its higher carbon and water-use efficiency (Vick and Young 2009). Nilsen *et al.* (1993) suggested that the carbon sequestration in branches during different seasons is the biochemical basis for their successful invasion of disturbed habitats. Non-photosynthetic organs containing chlorenchyma have greater respiration rates, which might be an important factor for understanding and explaining the interspecific water-use variations in this study. The trees with stem photosynthesis could generate more oxygen (O_2_) for respiration under light conditions than the species without green tissue on the trunk bark, even though the amount of oxygen is small. Since *C. hystrix* and *M. macclurei* have chlorenchyma on their stem barks (Fig. 7), they produced extra oxygen and had higher respiratory rates, supporting their occupancy of the dominant position.

Although the leaves are the main sites of photosynthesis, the trunk green tissue has the advantage of proximity by producing the carbohydrates for maintaining and repairing the xylem hydraulic system (Steppe *et al.* 2015; Bloemen *et al.* 2016). This might be an important factor for understanding and explaining not only the night-time water use, but also that the percentage of *Q*_n_/*Q*_d_ of photosynthetic stem species (*C. hystrix* and *M. macclurei*) was greater than those of non-photosynthetic stem species (*S. superba*).

## Conclusions

In this study, both the daytime and nocturnal water-use characteristics of three co-occurring woody tree species were studied in a low subtropical secondary forest. It was concluded that the intraspecific water use varied more than the interspecific water use. The meteorological factors, including VPD and air temperature, influenced the daytime sap flux, but meteorological factors (including VPD, wind speed and air temperature) had only trivial impacts on the night-time sap flux, meaning that night-time water use was mainly for water recharge in these three co-occurring species. The night-time replenishment of the trunk water deficit was correlated with the maximum night-time sap flux density (*J*_smax,n_). The night-time water use made a negligible contribution to the canopy transpiration. The results also revealed that the night-time water use and the percentage of *Q*_n_/*Q*_d_ of photosynthetic stem species (*C. hystrix* and *M. macclurei*) were greater than those of non-photosynthetic stem species (*S. superba*).

## Sources of Funding

This study was financially supported by the National Key Research and Development Programme (2016YFC0500106-02) and the National Natural Science Foundation of China (grant nos. 41630752, 31670410).

## Contributions by the Authors

Q.W., J.G.G., P.Z., L.W.Z., L.O., G.Y.N. and X.H.Z. designed research, performed research, analysed data and wrote the paper.

## Conflict of Interest

None declared.
